# Urinary, bowel and sexual symptoms in a cohort of patients with Friedreich’s ataxia

**DOI:** 10.1186/s13023-017-0709-y

**Published:** 2017-09-26

**Authors:** Meher Lad, Michael H. Parkinson, Myriam Rai, Massimo Pandolfo, Petya Bogdanova-Mihaylova, Richard A. Walsh, Sinéad Murphy, Anton Emmanuel, Jalesh Panicker, Paola Giunti

**Affiliations:** 1 Department of Molecular Neuroscience, Ataxia Centre, UCL Institute of Neurology and National Hospital for Neurology & Neurosurgery, Queen Square, London, WC1N 3BG UK; 2 Department of Uro-Neurology, National Hospital for Neurology and Neurosurgery and UCL Institute of Neurology, Queen Square, London, UK; 3 Department of Neurology, Hôpital Erasme, Université Libre de Bruxelles, Brussels, Belgium; 4 Department of Neurology, Adelaide & Meath Hospitals incorporating the National Children’s Hospital, Tallaght, Dublin, 24 Ireland; 5 Academic Unit of Neurology, Trinity College, Dublin, Ireland; 6 Department of Gastroenterology, University College London Hospitals, London, UK

**Keywords:** Friedreich’s ataxia, Urinary, Bladder, Bowel, Sexual function

## Abstract

**Background:**

Pelvic symptoms are distressing symptoms experienced by patients with Friedreich’s Ataxia (FRDA). The aim of this study was to describe the prevalence of lower urinary tract symptoms (LUTS), bowel and sexual symptoms in FRDA.

**Methods:**

Questionnaire scores measuring LUTS, bowel and sexual symptoms were analysed with descriptive statistics as a cohort and as subgroups (Early/Late-onset and Early/Late-stage FRDA) They were also correlated with validated measures of disease severity including those of ataxia severity, non-ataxic symptoms and activities of daily living.

**Results:**

80% (*n* = 46/56) of patients reported LUTS, 64% (*n* = 38/59) reported bowel symptoms and 83% (*n* = 30/36) reported sexual symptoms. Urinary and bowel or sexual symptoms were significantly likely to co-exist among patients. Late-onset FRDA patients were also more likely to report LUTS than early-onset ones. Patients with a longer disease duration reported higher LUTS scores and poorer quality of life scores related to urinary symptoms.

**Conclusions:**

A high proportion of FRDA have symptoms suggestive of LUTS, bowel and sexual dysfunction. This is more marked with greater disease duration and later disease onset. These symptoms need to be addressed by clinicians as they can have a detrimental effect on patients.

## Background

Friedreich’s Ataxia (FRDA) is the most common hereditary ataxia with an estimated prevalence in Western European populations varying from 1:125,000 to 1:20,000 [1, 2]. It is an autosomal recessive neurodegenerative disorder involving defects of the frataxin (*FXN*) gene on chromosome 9, which encodes the mitochondrial protein, frataxin [3]. This results in a deficiency of mitochondrial Complex I ﻿activity and an increase of lipid peroxidation causing cell death [4]. Its clinical phenotype includes neurological features such as impaired co-ordination, dysarthria, weakness, ocular ataxia, spasticity, sensory loss and hearing impairment, and non-neurological features such as cardiomyopathy, diabetes mellitus, kyphoscoliosis and foot deformities [5–7]. Atypical and much rarer phenotypes have also been described [8]. Less commonly studied features of FRDA include cognitive impairment and lower urinary tract symptoms (LUTS) [9].

Previous cohorts of FRDA patients have noted complaints of sphincter disturbance or urinary incontinence but this has not been clarified further [5, 10–13]. Only one large study of 258 FRDA patients showed that 82% of patients reported LUTS and 22% related these symptoms to poorer quality of life [14]. Smaller case-series have evaluated LUT function in a select group of patients but this has not been extended to larger groups. The aim of this study was to describe LUTS, bowel and sexual symptoms in FRDA patients and to describe this in relation to other established clinical and genetic features. A further aim was to look at the differences between early-onset and late-onset FRDA (more than 25 years of age) and early and late-stage FRDA (more than 15 years’ disease duration). Additionally, the co-existence of urinary, bowel and sexual symptoms and associations with clinical characteristics was studied.

## Methods

### Patient information and data collection

Patients (>16 years of age) with FRDA were recruited for this study. All questionnaires were administered at the time of an annual patient clinic appointment or returned at a later date via post. Informed consent was obtained from all patients and approval was gained from the local Research Ethics Committee as a service evaluation.

All patients had a confirmed diagnosis of FRDA by genetic analysis with homozygous expansions of intron 1 of the *FXN* gene. GAA repeat sizes were determined by polymerase chain reaction (PCR) by the Laboratoire de Neurologie Expérimentale of the Université Libre de Bruxelles (ULB) in Brussels, Belgium and the GAA repeat size of the smaller allele (GAA1) was used for further analysis.

### Questionnaires

The Scale for the Assessment and Rating of Ataxia (SARA) has been validated for both spinocerebellar ataxia and FRDA and has been used as a primary outcome measure in some studies [15, 16]. This quantifies ataxia symptoms with higher scores reflecting severe ataxia. The maximum score is 40. The Inventory of Non-Ataxic Symptoms (INAS) is a checklist of non-ataxic symptoms such as other motor and sensory symptoms, ocular signs and cognitive impairment [17]. This also includes a point for urinary dysfunction which was excluded from the questionnaire for our study. The maximum score is 29. The Activities of Daily Living (ADL) questionnaire was used as a marker of disability as it contains questions pertaining to daily function [18]. It is a section of the Friedreich Ataxia Rating Scale (FARS) [19]. The maximum score is 32. Patients did not complete the other sections of the FARS as they had already completed the questionnaires assessing disease severity as part of the European Friedreich’s Ataxia Consortium for Translational Studies (EFACTS) protocol [20].

The Urinary Symptom Profile (USP) was used to characterise urinary symptoms [21]. This is divided into 3 sections – Stress Incontinence (SI), Overactive Bladder (OAB) and Low Stream (LS) symptoms. OAB and LS scores were used for further analysis as these have been considered neurogenic symptoms. The maximum score is 21 for OAB and 7 for LS. We also included the 8-item SF-Qualiveen questionnaire which has been validated for assessing the quality of life in neurological patients with urinary symptoms [22]. This questionnaire focusses on 4 aspects of patients’ lives with respect to their urinary symptoms. This includes how frequently patients are affected and/or limited by their urinary symptoms and explores their fears and feelings with respect to this. The total maximum score is 4. The Neurogenic Bowel Dysfunction (NBD) score was also used to assess bowel symptoms in these patients [23]. This is a questionnaire comprising 10 questions regarding elements of bowel dysfunction: the instrument has been correlated with patient quality of life, specifically in patients with neurological disease. The categories in order of severity are: 0–6 ‘very minor’, 7–9 ‘minor’, 10–13 ‘moderate’ and >13 ‘severe’. The maximum score is 30. The Arizona Sexual Experience Scale (ASEX) was used to assess sexual symptoms in our patients [24]. This is a 5-item rating scale that has been validated and is widely used in the field of psychiatry. A patient is said to have sexual dysfunction if they score 19, a score of five in any one question or three fours in separate questions. The sexual domains consist of sexual drive, arousal, penile erection/ vaginal lubrication, orgasm and enjoyment.

### Statistical analysis

Descriptive analysis was conducted to calculate the mean, range (R) and standard deviation (SD) of the patient demographics, clinical characteristics and scores from the questionnaires evaluating their pelvic symptoms. This included a test of normality using the Shapiro-Wilks test. For this study, patients were also divided into those with a longer disease duration (>15 years) and those with shorter disease duration and into those with disease onset later in life (after 25 years of age) and those with young-onset FRDA. Group-level comparisons of mean values was performed using *t*-tests. To study the co-existence of urinary, bowel and sexual symptoms, an analysis of proportions of those patients that scored any points on the OAB, LS and NBD questionnaires and/or had sexual dysfunction on the ASEX was conducted using McNemar’s chi square test. This analyses differences in paired proportions from the same sample. Group-level analysis for patients that scored on any questionnaire and those that did not were performed using the Mann-Whitney U-test for non-parametric data and *t*-tests for parametric data. Pearson’s coefficients are shown for any correlations. This was performed to calculate the strength of association between variables related to disease severity and questionnaire scores for pelvic symptoms.

All statistical tests were conducted using IBM SPSS Statistics for Windows, version 21.0 (IBM Corp., Armonk, NY, 2012). A *p*-value of less than 0.05 was required for significance with Bonferroni correction.

## Results

### Patient Characteristics & Measures of disease severity

Fifty-nine patients (31 male) were recruited with a mean age of 35 years (*R* = 17–70, SD = 13). The mean age of onset of ataxia was 17 years (*n* = 53, R = 1–56, SD = 12) and mean duration of ataxia was 19 years (*n* = 50, *R* = 4–46, SD = 9). The mean size of the GAA (allele 1) repeats in the *FXN* gene was 642 repeats (n = 50, *R* = 67–1200, SD = 304) and for allele 2 was 944 (n = 50, *R* = 500–1234, SD = 218). Twenty-four patients were wheelchair bound. The signs elicited by our patients are shown in Table 1. SARA scores for the FRDA patients had a mean of 23 (*R* = 4.5–40, SD = 10), ADL scores had a mean of 16 (*R* = 1–32, SD = 9) and the INAS had a mean of 5 (*R* = 1–9, SD = 1.6). Results of these measures are shown in Table 2.

**Table 1:** Signs and Symptoms of FRDA patients in our Cohort (*n* = 59)

Clinical Feature	Percentage (%)
Gait Ataxia	100
Limb Ataxia	100
Dysarthria	90
Reduced Vibration Sense	88
Lower-limb Areflexia	78
Lower-limb Muscle Weakness	76
Swallowing Difficulties	67
Extensor Plantar Reflexes	56
Lower-limb Wasting	46
Horizontal Nystagmus	40
Saccadic Pursuit Eye Movements	8
Patellar Reflexes Present	6

*FRDA* – Friedreich’s Ataxia

**Table 2:** FRDA Patient Scores on Questionnaires

Questionnaire	*n = 59*	Mean Score	sd	Range
Age of Onset		16.8 years	12.3	1–56
Duration of Ataxia		18.6 years	9	4–46
SARA		23	10	4.5–40
ADL		16	9	1–32
INAS		5	1.6	1–9
Questionnaire	n*	Mean Score	sd	Range
OAB	56	4.3	4	0–12
SF-Qual	38	1.2	0.9	0.1–3.6
NBD	59	3	5	0–26
ASEX	36	10	7	0–36

*FRDA* – Friedreich’s Ataxia; *SARA* – Scale for the Assessment and Rating of Ataxia; *ADL* – Activities of Daily Living scale; *OAB* – Overactive Bladder score (subsection of Urinary Symptom Profile); *SF-Qual* – SF-Qualiveen quality of life score; *NBD* – Neurogenic Bowel Disease scale; *ASEX* – Arizona Sexual Experience scale. Higher scores indicate severe symptoms in each questionnaire

*The total number of patients is calculated based on those who replied to the specific questions in the questionnaires and were included in the final analysis

### LUTS, bowel and sexual symptoms scores

Forty-six patients (80%, *n* = 46/56) scored at least 1 point on the OAB section of the USP questionnaire and 17 (30%, *n* = 17/56) had LS symptoms. A breakdown of the OAB symptom scores is shown in Table 3. The OAB section had a mean score of 4.3 (*R* = 0–12, SD = 4). All patients scored at least 1 point on the SF-Qualiveen questionnaire (38 patients (68%, *n* = 56) completed the questionnaire). Scores had a mean of 1.2 (*R* = 0.1–3.6, SD = 0.9). The scores can be divided into the following categories with the percentage who reported symptoms in parenthesis: bother of limitations (73%), fears (89%), associated feelings (73%) and frequency of limitations (89%). Seven patients were taking anti-muscarinic medication for their urinary symptoms. One had had surgery for their symptoms, two required the use of a urinary catheter and one wore pads. These patients were excluded from some analyses as they did not complete most sections of the OAB and LS. Urodynamic studies in one patient showed detrusor overactivity and the maximum cystometric capacity in this patient was 350 mls. Another had urodynamic studies showing detrusor overactivity. Two patients had a mean residual volume of 30 mls (*R* = 20–40).

**Table 3:** Results from USP for OAB and LS symptoms

USP Section		*N* = 56*	Percentage (%)
OAB Score		45	80
	Frequency	42	75
	Urgency	33	59
	Incontinence	28	50
	Polyuria	19	34
	Nocturia	17	30
	Nocturnal Incontinence	7	13
LS Score		17	30
	Voiding	4	7
	Urinary Flow	7	13
	Stream	17	30

*USP*- Urinary Symptoms Profile, *OAB* – Overactive Bladder, *LS* – Low Stream

*2 patients did not complete all sections of the questionnaire as they used a urinary catheter and 1 patient did not complete all sections of the questionnaire as she wore pads

Thirty-seven patients (64%, *n* = 38/59) scored at least a point on the NBD questionnaire (Table 4). These patients reported the following more frequently: reduced frequency (86%), associated medication use (24%) and faecal incontinence (16%). The mean NBD score was 3 (*R* = 0–26, SD = 5): 6 (10%) patients scored above 10 (indicative of moderate to severe bowel dysfunction). There was no difference in the means of ambulatory and wheelchair-bound patients in a post-*hoc* analysis with independent *t*-tests. ASEX scores had a mean of 10 (*n* = 36, R = 0–30, SD = 7) (Table 5). According to the scoring criteria described above, 25% (*n* = 9, 5 females) had sexual dysfunction. One female and 1 male participant scored above 19. Seventeen out of the 36 patients that completed the questionnaire were sexually active. A summary of the total scores of the OAB, SF-Qual, NBD and ASEX questionnaires is given in Table 2.

**Table 4:** A Breakdown of Scores from the NBD Questionnaire

NBD Score		N = 59	Percentage (%)
NBD Combined		37	64
	Very Minor Dysfunction	28	48
	Minor Dysfunction	3	5
	Moderate Dysfunction	3	5
	Severe Dysfunction	3	5

*NBD*- Neurogenic Bowel Dysfunction Score

**Table 5:** ASEX Questionnaire sub-sections

ASEX Score		N = 36*	Percentage (%)
Sexually Active		17	47
Sexual Dysfunction		9	25
	>19 overall	1	3
	3 scores of 4	6	17
	5 in 1 domain	2	6

*ASEX*- Arizona Sexual Experience Scale

*23 out of 59 patients did not wish to complete the questionnaire

### Co-existing pelvic symptoms and severity of FRDA

Out of 45 patients who reported OAB, 33 (73%) also scored on the NBD questionnaire, reporting symptoms suggestive of bowel dysfunction (*p* = 0.12, McNemar’s chi-squared test) and those who had LS symptoms were significantly likely to have scored on the NBD (*n* = 14/17, *p* = 0.001, McNemar’s chi-squared test). These patients were likely to have higher scores in the NBD questionnaire (U = 143.5, *p* = 0.005) and ADLs (U = 162.0, *p* = 0.04) but not on the SARA and INAS. Patients with OAB were also significantly more likely to have scores on the ASEX indicating sexual dysfunction (*n* = 7/19, *p* < 0.001, McNemar’s chi-squared test). There were no patients without LUTS who had scores suggestive of sexual dysfunction (defined by considering all criteria) in our cohort. Patients with bowel symptoms were also more likely to have sexual dysfunction than not (*n* = 5/17, *p* = 0.021, McNemar’s chi-squared test).

### Relationship of duration of ataxia, measures of Disease Severity & Symptom Scores

The number of GAA repeats of the smaller allele was correlated with the onset of ataxia (*p* < 0.001, R(53) = −0.501) and SARA scores (*p* = 0.004, R(50) = 0.404) only. Duration of ataxia correlated significantly with increased scores on the ADLs questionnaire (*p* = 0.001, R(50) = 0.440) and with SARA scores (*p* = 0.006, R(50) = 0.383). This also showed significant correlations with OAB (*p* = 0.027, R(47) = 0.322) and LS scores (*p* = 0.014, R(48) = 0.353). A trend was observed with age of ataxia onset and OAB (*p* = 0.240, R(49) = 0.171) and LS (*p* = 0.108, R(50) = 0.230) scores.

Patients with a late-stage FRDA (defined as disease duration >15 years) had significantly higher scores of OAB by 2.5 points (t(45) = 2.24, *p* = 0.03), LS by 1 point (U = 180.0, *p* = 0.010), SF-Qualiveen by 0.8 points (U = 65.0, *p* = 0.009), SARA by 6.7 points (t(48) = 2.61, *p* = 0.012), ADLs by 6 points (t(48) = 2.74, *p* = 0.009) and INAS by 1 point (t(47) = 2.24, *p* = 0.03). Late-onset FRDA (defined as onset >25 years of age) patients had significantly higher scores in the OAB (t(47) = 2.53, *p* = 0.015) and LS scores by 1.4 points (t(48) = 3.07, *p* = 0.004).

## Discussion

To our knowledge, this is the first study to describe LUTS, bowel and sexual symptoms in a cohort of FRDA patients. Our findings show that a large proportion of patients report LUTS (80%) and bowel (64%) symptoms. 25% of patients are classified as having sexual dysfunction according to ASEX criteria. In the literature, other studies have reported the prevalence of ‘sphincter disturbance’ and ‘urinary incontinence’. This ranges from 7% to 41% but has not been explored further [5, 10–12, 25]. At a group level, we also showed significant differences in LUTS scores between early and late-onset FRDA patients. The latter had higher LUTS scores. The late-stage FRDA patients also had higher scores in this domain alongside higher scores on the SF-Qualiveen, indicating poorer urinary quality of life. This may be due to higher levels of spasticity in these patients or it may be physiological at an older age [20].

LUTS were commonly reported by our cohort of patients, however, only 24% of patients were on any form of treatment at presentation. The most commonly used agents for this were anti-muscarinic medications. The urodynamic findings were consistent with larger case-series that have shown detrusor overactivity as the predominant finding, however, only 2 patients had these tests [26, 27]. Other findings on urodynamic studies have included detrusor underactivity, detrusor acontractility and detrusor-sphincter dyssynergia [14]. This would account for the urinary disturbances reported by our patients as there were high scores in OAB indicating detrusor overactivity and LS indicating possible detrusor-sphincter dyssynergia due to pyramidal tract involvement or peripheral neuropathy.

The mechanisms for urinary disturbances in FRDA are multi-factorial, as disease affects the pyramidal tracts, posterior cord, spinocerebellar pathways, spinal ganglia, cerebellum and peripheral nerves [26, 28]. Lesions of the supra-pontine or spinal pathways that regulate LUT function affect the storage phase, causing reduced bladder capacity and detrusor overactivity. Spasticity has been well described in FRDA patients, especially in the later stages of the disease [20]. However, our findings did not correlate significantly with spasticity scores after correction for multiple comparisons. This is possibly due to the severe peripheral neuropathy that ‘masks’ corticospinal tract involvement. Additionally, although patients with peripheral neuropathy report sensation of bladder fullness, inability to initiate micturition voluntarily and bladder distension, a subset experience detrusor overactivity, which could be a result of bladder decentralisation due to preserved peripherally sited post-ganglionic neurons [29].

Neuropathological studies of FRDA have shown degeneration of the dentate nuclei, dorsal root ganglia, peripheral nerves and corticospinal tracts [30]. Neurophysiological studies reveal that formerly quiescent C-fibres become the major afferent from the bladder, after spinal cord injury, which drives detrusor overactivity via activation of this aberrant spinal reflex [31]. Spinal pathways connecting the pontine micturition centre to the sacral cord coordinate reciprocal activity of the detrusor and sphincter muscles. Interruption of these pathways results in detrusor-sphincter dyssynergia causing incomplete bladder emptying with high detrusor pressures. Sacral and infra-sacral lesions lead to poorly sustained detrusor contractions and/or non-relaxing sphincters and damage to parasympathetic innervation of the detrusor and bowel, which is responsible for accelerating bowel transit, can lead to urinary incontinence and constipation respectively [32]. Similarly, degeneration of the somatic and parasympathetic nervous system may give rise to symptoms suggestive of sexual dysfunction [33].

More than half of our patients (64%) reported symptoms suggestive of neurogenic bowel dysfunction albeit the mean score indicated ‘very minor’ bowel dysfunction. Nevertheless, there was a significant minority of patients with moderate or severe bowel symptomatology, suggesting that for some, bowel dysfunction can be an extremely intrusive aspect of FRDA. We noted that 25% of our FRDA patients who completed the ASEX questionnaire had sexual dysfunction. This was not confined to any domain as patients reported high scores diagnostic of sexual dysfunction uniformly for all questions. In addition, we did not find any significant relationships with rating scales of FRDA severity and this may be due to the complex behavioural and neurophysiological nature of sexual function.

Patients with urinary or bowel symptoms were significantly more likely to have sexual dysfunction that those without. This highlights the importance for screening for symptoms in all three domains when patients are encountered by clinicians. When urinary symptoms are encountered, for example, one can manage this with medication such as antimuscarinic agents, intravesical Botox injections, percutaneous tibial nerve stimulation and/or referral to specialist urology services. Moreover, patients were significantly affected in terms of their quality of life for urinary symptoms and we would anticipate similar ratings in quality of life measures for bowel and sexual symptoms. Studies in the future need to address these issues. Some limitations of our study include the slightly older cohort of patients compared to other large studies and the lack of neuropathological data. Urodynamic data on all patients would also be helpful to characterise any differences in sub-groups.

## Conclusions

LUTS, bowel and sexual symptoms are under-recognised, under-discussed and undertreated. Clarifying whether these symptoms are related to pelvic dysfunction will also allow clinicians to target therapies to manage these highly complex patients in a multi-disciplinary setting.
